# Comparative study on the effect of elevated levels of carbon dioxide on growth and photosynthesis of three selected plant species

**DOI:** 10.3389/fpls.2026.1772837

**Published:** 2026-05-15

**Authors:** Abdelghafar M. Abu-Elsaoud, Eman Obaid Aldawsari, Reem Nigr Alotaibi, Abeer abdulrahman Aldayel, Alya Nasser Abdulrahman Alarefi, Renad Mohammed Salem Aldossari, Seham M. Al Raish

**Affiliations:** 1 Department of Biology, College of Science, Imam Mohammad Ibn Saud Islamic University (IMSIU), Riyadh, Saudi Arabia; 2 Country Department of Biology, College of Science, United Arab Emirates University, Al Ain, United Arab Emirates

**Keywords:** Elevated CO_2_, growth, photosynthesis, C3, C4, *Capsicum annuum*, *Mentha × piperita*, *Gomphrena globosa*

## Abstract

The aim of this study was to evaluate the growth and physiological responses of two C3 species (*Capsicum annuum* and *Mentha × piperita*) and one C4 species (*Gomphrena globosa*) under elevated CO_2_ conditions. Plants were exposed to three CO_2_ concentrations—~400 parts per million (ppm) (control), ~2,900 ppm, and ~5,400 ppm—in a controlled greenhouse experiment. Elevated CO_2_ significantly enhanced plant height, leaf number, bud formation, shoot and root fresh weight, total biomass, chlorophyll content, and relative leaf nitrogen index across all species. The magnitude of response differed among species, with *C. annuum* showing the strongest overall growth stimulation at 5,400 ppm. Multivariate analyses further confirmed that CO_2_ concentration significantly influenced physiological parameters. These findings demonstrate species-specific responses of C3 and C4 plants to high CO_2_ enrichment and highlight potential implications for crop productivity under changing atmospheric conditions.

## 1 Introduction

Atmospheric carbon dioxide (CO_2_) concentrations have increased substantially due to anthropogenic activities and are projected to continue to rise throughout this century, with significant implications for global climate and agricultural productivity (Sage, 1994; Cassia et al., 2018; Shishegaran et al., 2020; Wang et al., 2022; Sendall et al., 2024; Jiang et al., 2025). Because CO_2_ is the primary substrate for photosynthesis, rising atmospheric CO_2_ directly influences plant carbon assimilation, biomass production, and ecosystem carbon cycling (Sage, 1994; Keenan et al., 2022). In addition to their ecological and agronomic importance, plant species, particularly those with medicinal properties, are critical components of global healthcare systems, with growing evidence of widespread reliance on plant-based remedies and strong associations among knowledge, perceptions, and patterns of use (Almasri et al., 2025; Bedir et al., 2025; Al Raish et al., 2025, 2026; Al Raish, 2026). Global photosynthesis represents the largest CO_2_ flux in the carbon cycle, and even modest changes in photosynthetic performance can substantially alter carbon balance and crop productivity (Keenan et al., 2022; Baslam and Sanz-Saez, 2023). Although elevated CO_2_ generally enhances net photosynthesis in C3 species, long-term exposure often induces acclimation responses characterized by reductions in maximum carboxylation capacity (Vcmax), ribulose-1,5-bisphosphate carboxylase/oxygenase (RuBisCO) content, and leaf nitrogen concentration (Ainsworth and Long, 2021; Ancín et al., 2024). These acclimatory adjustments complicate predictions of future crop performance under elevated CO_2_ scenarios.

Plants utilize two primary photosynthetic pathways, C3 and C4, which differ fundamentally in their carbon fixation mechanisms and responses to atmospheric CO_2_ enrichment (Raines, 2011; Cheng and He, 2019; Verhage, 2021; Chen et al., 2023; Mukundan et al., 2024). In C3 species, CO_2_ is directly fixed by RuBisCO, a process accompanied by photorespiration that reduces carbon-use efficiency under ambient CO_2_ (Raines, 2011; Mukundan et al., 2024). Elevated CO_2_ suppresses photorespiration in C3 plants, typically enhancing photosynthetic carbon gain and biomass accumulation (Bravdo and Canvin, 1979; Zheng et al., 2019; Busch, 2020). In contrast, C4 plants possess a CO_2_-concentrating mechanism that minimizes photorespiration under current atmospheric conditions, resulting in comparatively smaller stimulation under elevated CO_2_ (Verhage, 2021; Mukundan et al., 2024). Large-scale syntheses and recent experimental studies confirm that C3 crops generally exhibit stronger growth and photosynthetic stimulation than C4 species under elevated CO_2_, although responses vary across environmental conditions and developmental stages (Reich et al., 2018; Kaur et al., 2025).

Beyond steady-state photosynthesis, elevated CO_2_ also alters stomatal conductance, biochemical capacity, and the balance between diffusional and biochemical limitations (Ancín et al., 2024; Tang et al., 2024). Recent work demonstrates that elevated CO_2_ can shift the dominant limitation of photosynthesis from stomatal to biochemical constraints during induction, depending on species-specific stomatal morphology and resource allocation patterns (Tang et al., 2024; Zhang et al., 2024). Moreover, interactions between elevated CO_2_ and environmental factors such as temperature, drought, and nutrient availability further influence RuBisCO regulation, electron transport capacity, and nitrogen dynamics (Wang et al., 2022; Ancín et al., 2024). These findings underscore the importance of evaluating species-specific physiological plasticity under controlled CO_2_ enrichment conditions.

Although numerous studies have examined the effects of elevated CO_2_ on individual crop species, direct comparative evaluations of C3 and C4 plants under identical controlled high-CO_2_ exposure remain limited. Such comparisons are critical for understanding pathway-dependent physiological responses, nitrogen-related adjustments, and biomass allocation patterns that may influence future crop selection and adaptation strategies.

In this study, two C3 species (*Capsicum annuum* and *Mentha × piperita*) and one C4 species (*Gomphrena globosa*) were selected to investigate differential physiological responses under controlled CO_2_ enrichment. These species were chosen based on both physiological and practical considerations: *C. annuum* and *M. piperita* are economically important C3 crops, while *G. globosa* represents a C4 photosynthetic pathway. This combination enables direct comparison of growth, chlorophyll dynamics, and relative nitrogen status between photosynthetic types under strong CO_2_ enrichment conditions.

The selected plant species were chosen based on both physiological and practical considerations. *C. annuum* (pepper) and *Mentha × piperita* (peppermint) are economically important C3 crops widely cultivated for food and medicinal uses. *G. globosa*, a representative C4 species, was included to allow direct comparison between C3 and C4 photosynthetic pathways under identical CO_2_ enrichment conditions. This combination enables evaluation of pathway-dependent growth and physiological responses relevant to agricultural adaptation under rising atmospheric CO_2_.

The objectives of this study were to:

1. evaluate the growth responses of two C3 species (*C. annuum* and *Mentha × piperita*) and one C4 species (*G. globosa*) under elevated CO_2_ concentrations [400, 2,900, and 5,400 parts per million (ppm)];
2. assess changes in chlorophyll content, relative leaf nitrogen index, and biomass allocation under high CO_2_ exposure; and
3. compare species-specific physiological responses between C3 and C4 photosynthetic pathways under controlled CO_2_ enrichment conditions.

The selection of plant species in this study was deliberate to enable a comparative evaluation of different photosynthetic pathways under elevated CO_2_ conditions. *C. annuum* and *Mentha × piperita* are C3 species, while *G. globosa* follows the C4 photosynthetic pathway. Since C3 plants generally exhibit stronger stimulation of photosynthesis under elevated CO_2_ due to reduced photorespiration, whereas C4 plants already possess a CO_2_-concentrating mechanism, comparing these species provides insight into pathway-specific growth and physiological responses under high CO_2_ environments.

Although the stimulatory effects of elevated CO_2_ on plant growth have been widely reported, comparative studies integrating C3 and C4 species under identical experimental conditions and high CO_2_ exposure remain limited. The novelty of the present study lies in the simultaneous evaluation of two C3 crops and one C4 species using a unified experimental system, combined with comprehensive physiological, biochemical, and multivariate analyses. This approach allows a clearer interpretation of pathway-specific responses to elevated CO_2_ enrichment.

## 2 Materials and methods

### 2.1 Experimental design

**Figure 1:**
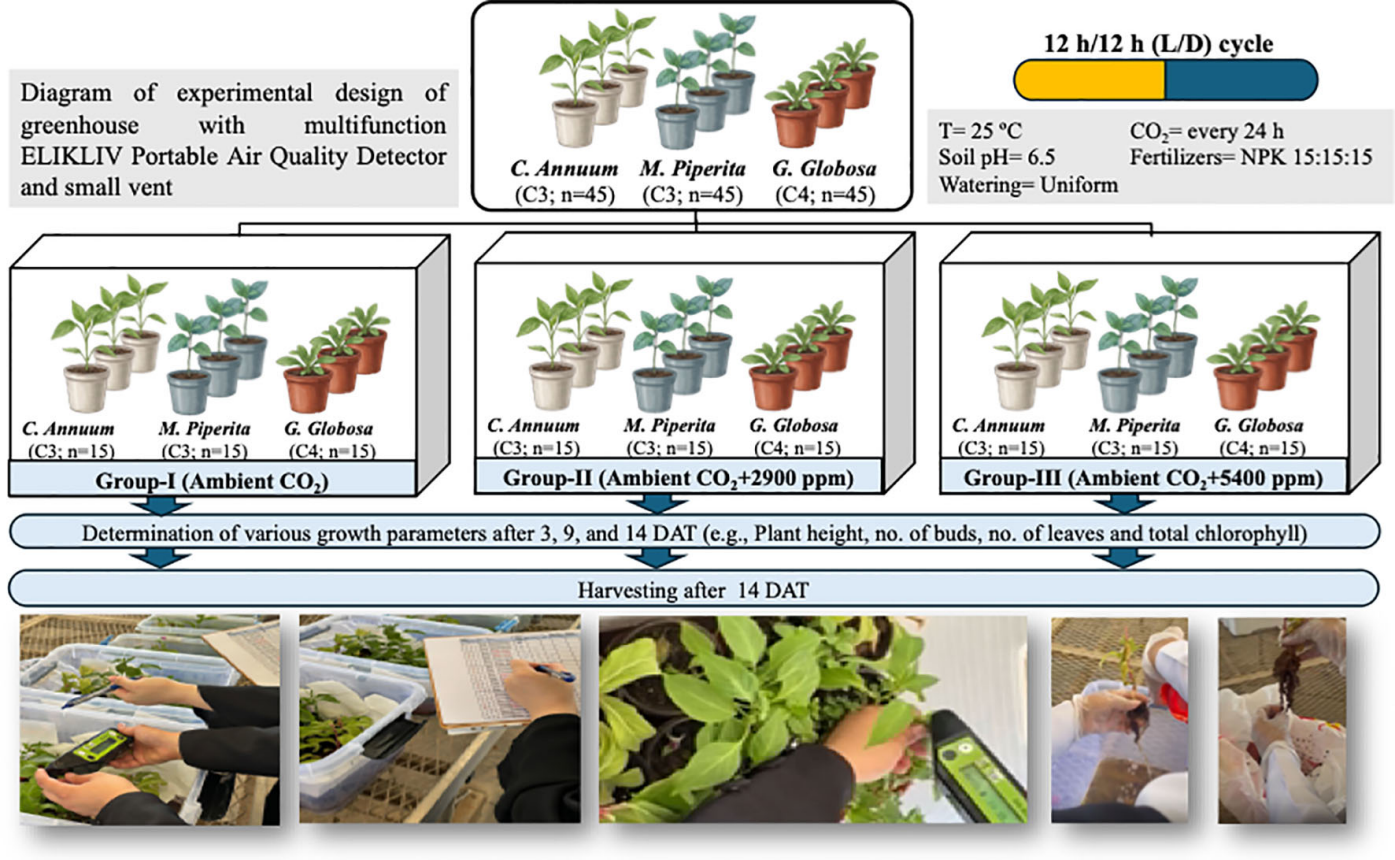
Experimental greenhouse setup and CO_2_ treatment design. The controlled environment chamber was equipped with a multifunction portable air quality detector (ELIKLIV) and a ventilation system to maintain target CO_2_ concentrations. A total of 45 plants per species (*Gomphrena globosa, Capsicum annuum,* and *Mentha × piperita*) were distributed equally across three CO_2_ treatment groups: Group I — ambient control (~400 ppm), Group II — elevated CO_2_ (~2,900 ppm), and Group III — highly elevated CO_2_ (~5,400 ppm). Representative photographs of the experimental conditions and plant materials are shown.

This study was conducted to evaluate the effects of elevated CO_2_ concentrations on the growth and photosynthesis of three selected plant species: *G. globosa*, *C. annuum*, and *M. piperita*. Each species was subjected to three different CO_2_ treatment levels over a specified period to assess the influence of elevated CO_2_ on various growth and physiological parameters (**Figure 1**).

Plants were grown in 7-cm-diameter pots filled with a uniform mixture of sterilized soil and peat moss (3:1 ratio). The soil had a pH of 6.5 and was supplemented with a balanced slow-release fertilizer (NPK 15-15-15) to ensure adequate nutrient supply throughout the experiment. Watering was conducted uniformly with tap water as needed to maintain optimal soil moisture levels across all experimental units. The experiment was carried out in a naturally lit greenhouse with a controlled temperature of 25 °C (± 2 °C), and plants were exposed to a 12-h photo period of natural sunlight filtered through transparent containers. A total of 45 plants of each species were divided into three treatment groups based on the CO_2_ concentration they were exposed to:

Group I (Control): Plants were exposed to ambient CO_2_ levels (~400 ppm), which were continuously monitored and maintained under natural greenhouse conditions throughout the experiment.
Group II: ~2,900 ppm.
Group III: ~5,400 ppm.

Each treatment group contained five replicates per species (*n* = 5 per species per treatment), totaling 15 plants per treatment and 45 plants per species. The methodology was implemented following the procedures described by Shenglei and Ferris (2006) and Akhlaq et al. (2023).

CO_2_ enrichment was achieved using dry ice (solid CO_2_) to simulate elevated atmospheric CO_2_ levels. Transparent plastic containers were used to enclose each treatment group, ensuring controlled CO_2_ concentrations while allowing sufficient light penetration. Dry ice was placed in small, perforated containers within each sealed chamber to gradually release CO_2_ through sublimation. The amount of dry ice required for each treatment was calculated based on the container volume and the desired CO_2_ concentration. For Groups II and III, dry ice quantities were calculated to achieve the target final chamber concentrations of approximately 2,900 and 5,400 ppm, respectively.

CO_2_ levels in all treatment groups, including the ambient control, were continuously monitored using a Multifunction ELIKLIV Portable Air Quality Detector (LORD Electrical, Shenzhen Yong An Cun Technology Co., Ltd, China) to ensure stability. To regulate CO_2_ release and prevent oxygen depletion, small vents were incorporated into the containers to allow excess gas to escape without significant CO_2_ loss. Dry ice was replenished every 24 h to maintain consistent CO_2_ concentrations, as the sublimation rate varied with temperature and container size.

All CO_2_ values reported in this study refer exclusively to the final monitored concentrations inside the experimental chambers.

Although the applied CO_2_ concentrations exceed projected near-future atmospheric levels, the purpose of this study was to examine short-term physiological and growth responses under strong CO_2_ enrichment conditions in a controlled greenhouse system. Such enrichment levels are commonly used in experimental plant physiology to amplify metabolic responses and allow clearer differentiation between C3 and C4 species.

### 2.2 Growth parameters

Growth and physiological parameters were measured at 3, 9, and 14 days after treatment (DAT) intervals throughout the experiment. The parameters assessed included the following:

1. Plant height (cm)
2. Number of leaves
3. Number of buds
4. Total leaf chlorophyll content (estimated using an AtLeaf device, FT Green LLC)
5. Relative leaf nitrogen index (estimated using the AtLeaf chlorophyll meter)
6. Root length (cm)
7. Shoot and root fresh weight (FW)

The AtLeaf device provided a non-destructive estimate of relative chlorophyll content for continuous monitoring throughout the study (**Supplementary Figures 1**-**4**).

Leaf nitrogen status was estimated using the AtLeaf chlorophyll meter (FT Green LLC, USA). The device provides relative index readings based on leaf chlorophyll measurements that are correlated with nitrogen status. The values reported in this study represent AtLeaf index units rather than direct chemical nitrogen concentration.

The present study focused exclusively on physiological and growth responses to elevated CO_2_ and did not include molecular analyses such as gene expression or enzymatic activity assays.

### 2.3 Statistical analysis

All collected data, including plant height, number of leaves, number of buds, chlorophyll content, relative leaf nitrogen index, root length, and shoot and root FW, were recorded in a spreadsheet for statistical analysis. Data normality was verified using the Shapiro–Wilk and Kolmogorov–Smirnov tests. Since the data followed a parametric distribution, results were presented as mean ± standard error of the mean (SEM).

A one-way analysis of variance (ANOVA) was performed to determine the effects of CO_2_ concentration on each parameter for all plant species. A Tukey’s HSD *post hoc* test was conducted to compare group differences. Statistical significance was determined at *p* < 0.05, *p* < 0.01, and *p* < 0.001. All analyses used IBM-SPSS software (Version 30.0 for Mac OS).

## 3 Results

Elevated CO_2_ significantly improved several physiological and biochemical traits, including plant height, leaf number, bud formation, shoot and root fresh weight, total biomass, chlorophyll content, relative leaf nitrogen index, and biomass allocation (shoot/root ratio).

### 3.1 Plant height

**Figure 2:**
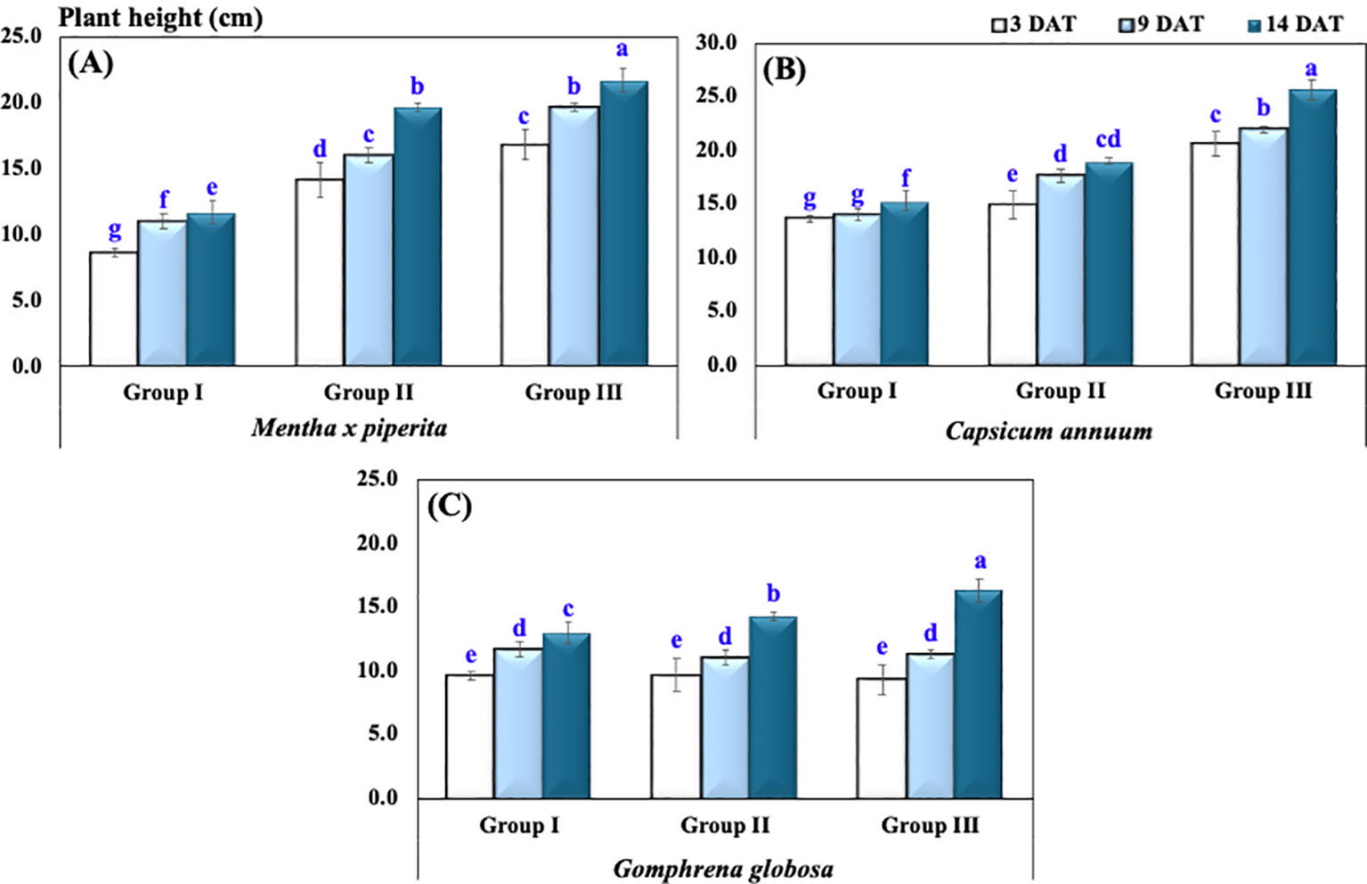
Plant height under varying CO_2_ concentrations in **(A)** *Mentha x piperita*, **(B)** *Capsicum annuum*, **(C)** *Gophrena globosa*. Different letters indicate statistically significant differences based on Tukey’s HSD test at *p* ≤ 0.05. Treatments included Group I (Control, ~400 ppm CO_2_), Group II (Elevated CO_2_, ~2,900 ppm), and Group III (Highly Elevated CO_2_, ~5,400 ppm). DAT, Days after treatment.

The plant height of *M. piperita* in Group I measured at 3, 9, and 14 DAT exhibited an average ± standard deviation (SD) of 8.7 ± 0.6, 11.0 ± 1.0, and 11.7 ± 1.5 cm, respectively. In Group II, the corresponding values were 14.2 ± 2.3, 16.0 ± 1.0, and 19.7 ± 0.6 cm, while in Group III, plant height increased to 16.8 ± 2.0, 19.7 ± 0.6, and 21.7 ± 1.5 cm at the same time points (**Table 1**, **Figure 2**).

**Table 1:** The plant height (cm) under varying CO_2_ concentrations presented as mean and standard deviation.

Plant species	Treatment group	Plant height		
		3 DAT	9 DAT	14 DAT
*Mentha × piperita*	Group I	8.7 ± 0.6 g	11.0 ± 1.0 f	11.7 ± 1.5 e
	Group II	14.2 ± 2.3 d	16.0 ± 1.0 c	19.7 ± 0.6 b
	Group III	16.8 ± 2.0 c	19.7 ± 0.6 b	21.7 ± 1.5 a
*Capsicum annuum*	Group I	13.7 ± 1.5 g	14.0 ± 1.0 g	15.3 ± 1.2 f
	Group II	15.0 ± 1.0 e	17.7 ± 0.6 d	19.0 ± 1.0 cd
	Group III	20.7 ± 1.2 c	22.0 ± 3.5 b	25.7 ± 0.6 a
*Gomphrena globosa*	Group I	9.7 ± 1.2 e	11.7 ± 0.6 d	13.0 ± 1.0 c
	Group II	9.7 ± 1.2 e	11.0 ± 1.0 d	14.3 ± 1.2 b
	Group III	9.3 ± 0.6 e	11.3 ± 1.2 d	16.3 ± 0.6 a
Two-way repeated-measures ANOVA				
Plant species		<0.001***		
Treatment		<0.001***		
Time		<0.001***		
Plant × Treatment × Time		<0.001***		

Significance levels are indicated as *p* < 0.05 (*), *p* < 0.01 (**), and *p* < 0.001 (***), while “ns” denotes non-significant differences (*p* > 0.05). Different letters (a, b) indicate statistically significant differences according to Tukey’s HSD test at *p* ≤ 0.05. Abbreviations: ANOVA, analysis of variance; DAT, days after treatment. CO_2_ treatments: Group I (Control, ~400 ppm CO_2_), Group II (Elevated CO_2_, ~2,900 ppm CO_2_), and Group III (Highly Elevated CO_2_, ~5,400 ppm CO_2_).

For *C. annuum*, plant height in Group I at 3, 9, and 14 DAT was recorded as 13.7 ± 1.5, 14.0 ± 1.0, and 15.3 ± 1.2 cm, respectively. In Group II, plant height increased to 15.0 ± 1.0, 17.7 ± 0.6, and 19.0 ± 1.0 cm, while in Group III, it reached 20.7 ± 1.2, 22.0 ± 3.5, and 25.7 ± 0.6 cm over the same period.

Moreover, for *G. globosa*, plant height showed a progressive increase across all groups over time. At 3 DAT, Group I and Group II recorded identical values of 9.7 ± 1.2 cm, whereas Group III was slightly lower at 9.3 ± 0.6 cm. By 9 DAT, plant height increased modestly in all groups, reaching 11.7 ± 0.6 cm in Group I, 11.0 ± 1.0 cm in Group II, and 11.3 ± 1.2 cm in Group III. The greatest increase was observed at 14 DAT, with Group III attaining the highest value (16.3 ± 0.6 cm), followed by Group II (14.3 ± 1.2 cm) and Group I (13.0 ± 1.0 cm), indicating that Group III promoted the strongest growth response over time.

A two-way ANOVA revealed a highly significant effect on the total number of plant leaves, influenced by plant species (*p* < 0.001), treatment groups (*p* < 0.001), and the interaction between species and treatment groups (*p* < 0.001).

### 3.2 Number of leaves

**Figure 3:**
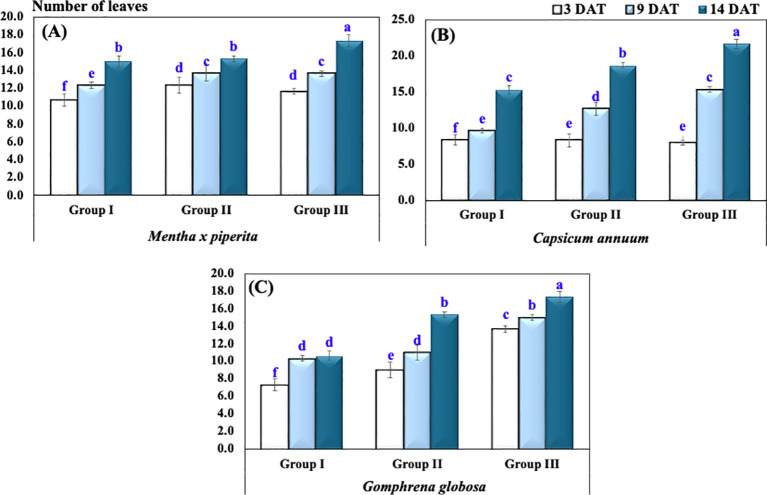
Number of leaves of plants under different CO_2_ concentrations in **(A)** *Mentha x piperita*, **(B)** *Capsicum annuum*, **(C)** *Gophrena globosa*. Different letters indicate significant differences based on Tukey’s HSD test at *p* ≤ 0.05. Treatments included Group I (Control, ~400 ppm CO_2_), Group II (Elevated CO_2_, ~2,900 ppm), and Group III (Highly Elevated CO_2_, ~5,400 ppm). DAT, Days after treatment.

The number of leaves in *M. piperita* at 3, 9, and 14 DAT in Group I exhibited an average (± SD) of 10.7 ± 1.2, 12.3 ± 0.6, and 15.0 ± 1.0, respectively. In Group II, the corresponding values were 12.3 ± 1.5, 13.7 ± 1.5, and 15.3 ± 0.6, while in Group III, the number of leaves increased to 11.7 ± 0.6, 13.7 ± 0.6, and 17.3 ± 1.2 (**Table 2**, **Figure 3**).

**Table 2:** The number of leaves under varying CO_2_ concentrations presented as mean and standard deviation.

Plant species	Treatment group	No. of leaves		
		3 DAT	9 DAT	14 DAT
*Mentha × piperita*	Group I	10.7 ± 1.2 f	12.3 ± 0.6 e	15.0 ± 1.0 b
	Group II	12.3 ± 1.5 d	13.7 ± 1.5 c	15.3 ± 0.6 b
	Group III	11.7 ± 0.6 d	13.7 ± 0.6 c	17.3 ± 1.2 a
*Capsicum annuum*	Group I	8.3 ± 0.6 f	9.7 ± 0.6 e	15.3 ± 0.6 c
	Group II	8.3 ± 0.6 e	12.7 ± 1.2 d	18.7 ± 1.2 b
	Group III	8.0 ± 1.0 e	15.3 ± 1.2 c	21.7 ± 1.5 a
*Gomphrena globosa*	Group I	7.3 ± 0.6 f	10.3 ± 0.6 d	10.7 ± 0.6 d
	Group II	9.0 ± 1.0 e	11.0 ± 1.0 d	15.3 ± 1.5 b
	Group III	13.7 ± 0.6 c	15.0 ± 1.0 b	17.3 ± 0.6 a
Two-way repeated-measures ANOVA				
Plant species		<0.001***		
Treatment		<0.001***		
Time		<0.001***		
Plant × Treatment × Time		<0.001***		

Significance levels are indicated as *p* < 0.05 (*), *p* < 0.01 (**), and *p* < 0.001 (***), while “ns” denotes non-significant differences (*p* > 0.05). Different letters (a, b) indicate statistically significant differences according to Tukey’s HSD test at *p* ≤ 0.05. Abbreviations: ANOVA, analysis of variance; DAT, days after treatment. CO_2_ treatments: Group I (Control, ~400 ppm CO_2_), Group II (Elevated CO_2_, ~2,900 ppm CO_2_), and Group III (Highly Elevated CO_2_, ~5,400 ppm CO_2_).

Similarly, for *C. annuum*, the number of leaves at 3, 9, and 14 DAT in Group I was recorded as 8.3 ± 0.6, 9.7 ± 0.6, and 15.3 ± 0.6, respectively. In Group II, the number of leaves increased to 8.3 ± 0.6, 12.7 ± 1.2, and 18.7 ± 1.2, while in Group III, it reached 8.0 ± 1.0, 15.3 ± 1.2, and 21.7 ± 1.5 over the same period.

For *G. globosa*, the number of leaves in Group I at 3, 9, and 14 DAT showed an average (± SD) of 7.3 ± 0.6, 10.3 ± 0.6, and 10.7 ± 0.6, respectively. In Group II, the values increased to 9.0 ± 1.0, 11.0 ± 1.0, and 15.3 ± 1.5, while in Group III, the corresponding values were 13.7 ± 0.6, 15.0 ± 1.0, and 17.3 ± 0.6.

A two-way ANOVA revealed a highly significant effect on the total number of plant leaves, influenced by plant species (*p* < 0.001), treatment groups (*p* < 0.001), and the interaction between species and treatment groups (*p* < 0.001).

### 3.3 Number of buds

**Figure 4:**
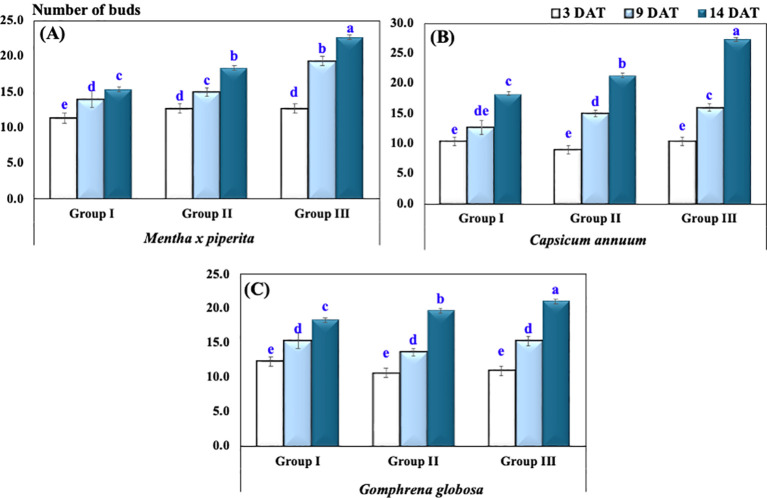
Number of buds of plants under varying CO_2_ concentrations in **(A)** *Mentha x piperita*, **(B)** *Capsicum annuum*, **(C)** *Gophrena globosa*. Different letters indicate significant differences based on Tukey’s HSD test at *p* ≤ 0.05. Treatments included Group I (Control, ~400 ppm CO_2_), Group II (Elevated CO_2_, ~2,900 ppm), and Group III (Highly Elevated CO_2_, ~5,400 ppm). DAT, Days after treatment.

The number of buds in *M. piperita* at 3, 9, and 14 DAT in Group I exhibited an average (± SD) of 11.3 ± 1.2, 14.0 ± 2.0, and 15.3 ± 0.6, respectively. In Group II, the corresponding values were 12.7 ± 1.2, 15.0 ± 1.0, and 18.3 ± 0.6, while in Group III, the number of buds increased to 12.7 ± 1.2, 19.3 ± 1.2, and 22.7 ± 0.6 (**Figure 4**).

Similarly, for *C. annuum*, the number of buds at 3, 9, and 14 DAT in Group I was recorded as 10.3 ± 1.5, 12.7 ± 1.2, and 18.3 ± 0.6, respectively. In Group II, the values increased to 9.0 ± 1.0, 15.0 ± 1.0, and 21.3 ± 1.5, while in Group III, they reached 10.3 ± 1.5, 16.0 ± 1.7, and 27.3 ± 2.3.

For *G. globosa*, the number of buds in Group I at 3, 9, and 14 DAT showed an average (± SD) of 12.3 ± 0.6, 15.3 ± 1.2, and 18.3 ± 2.5, respectively. In Group II, the values were 10.7 ± 1.2, 13.7 ± 1.5, and 19.7 ± 1.5, while in Group III, the corresponding values were 11.0 ± 1.0, 15.3 ± 2.5, and 21.0 ± 1.0 (**Figure 2**).

A two-way ANOVA revealed a highly significant effect of treatment groups (*p* < 0.001) and the interaction between treatment groups and species (*p* < 0.001) on the total number of buds. However, the difference in bud numbers among plant species was not statistically significant.

### 3.4 Total leaf chlorophyll content

**Figure 5:**
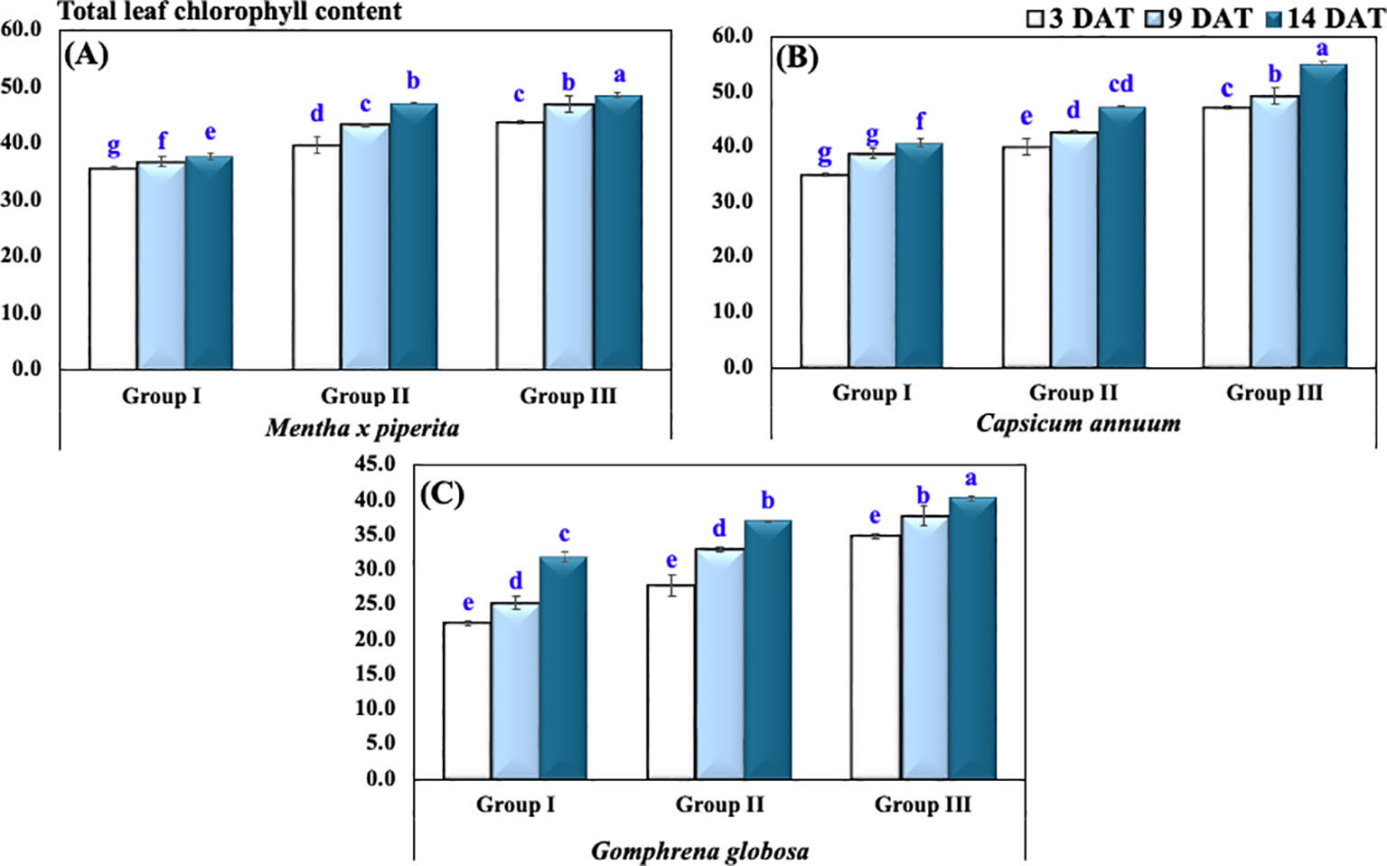
Total leaf chlorophyll content of plants under varying CO_2_ concentrations in **(A)** *Mentha x piperita*, **(B)** *Capsicum annuum*, **(C)** *Gophrena globosa*. Different letters indicate significant differences based on Tukey’s HSD test at *p* ≤ 0.05. Treatments included Group I (Control, ~400 ppm CO_2_), Group II (Elevated CO_2_, ~2,900 ppm), and Group III (Highly Elevated CO_2_, ~5,400 ppm). DAT, Days after treatment.

The chlorophyll content in *M. piperita* at 3, 9, and 14 DAT in Group I showed an average (± SD) of 35.5 ± 0.5, 36.7 ± 1.5, and 37.7 ± 1.2, respectively. In Group II, the corresponding values were 39.7 ± 2.5, 43.2 ± 0.6, and 47.2 ± 0.3, while in Group III, chlorophyll content increased to 43.7 ± 0.6, 46.9 ± 2.5, and 48.5 ± 0.6 (**Figure 5**).

Similarly, in *C. annuum*, total leaf chlorophyll content at 3, 9, and 14 DAT in Group I was recorded as 34.9 ± 0.6, 38.7 ± 1.2, and 40.8 ± 2.3, respectively. In Group II, the values increased to 39.9 ± 0.8, 42.6 ± 0.6, and 47.4 ± 0.4, while in Group III, they reached 47.1 ± 0.5, 49.2 ± 0.3, and 55.1 ± 1.5.

For *G. globosa*, chlorophyll content at 3, 9, and 14 DAT in Group I showed an average (± SD) of 22.4 ± 0.8, 25.3 ± 4.0, and 31.8 ± 1.4, respectively. In Group II, the values were 27.7 ± 1.9, 32.9 ± 6.1, and 36.9 ± 1.5, while in Group III, the corresponding values increased to 34.9 ± 3.5, 37.7 ± 2.0, and 40.3 ± 1.6.

A two-way ANOVA indicated a highly significant effect of plant species (*p* < 0.001), time of observation (*p* < 0.001), treatment groups (*p* < 0.001), and the interaction between treatment groups and species (*p* < 0.001) on total leaf chlorophyll content.

### 3.5 Relative leaf nitrogen index

The relative leaf nitrogen index in *M. piperita* exhibited a progressive increase across treatment groups. In Group I, the values at 3, 9, and 14 DAT were 94.9 ± 1.4, 98.2 ± 4.3, and 101.0 ± 3.3, respectively. In Group II, the corresponding values were 106.7 ± 7.1, 116.7 ± 1.7, and 127.9 ± 0.8. Group III showed the highest nitrogen content, with 118.0 ± 1.6, 127.1 ± 6.9, and 131.5 ± 1.7 at 3, 9, and 14 DAT, respectively.

Similarly, in *C. annuum*, relative leaf nitrogen index in Group I was recorded as 93.2 ± 1.7, 104.0 ± 3.4, and 109.8 ± 6.4 at 3, 9, and 14 DAT. In Group II, the values increased to 107.3 ± 2.2, 115.1 ± 1.7, and 128.5 ± 1.0. Group III exhibited the highest nitrogen content, with values of 127.6 ± 1.3, 133.6 ± 0.7, and 150.2 ± 4.3 at the respective time points.

For *G. globosa*, the relative leaf nitrogen index in Group I was 57.8 ± 2.3, 66.1 ± 11.3, and 84.6 ± 4.1 at 3, 9, and 14 DAT. In Group II, the recorded values were 73.0 ± 5.5, 87.6 ± 17.1, and 98.9 ± 4.2. Group III exhibited the highest nitrogen content, with values of 93.1 ± 9.9, 101.0 ± 5.7, and 108.4 ± 4.4 at the respective time points (**Figure 2**).

A two-way ANOVA indicated a highly significant effect of plant species (*p* < 0.001), time of observation (*p* < 0.001), treatment groups (*p* < 0.001), and the interaction between treatment groups and species (*p* < 0.001) on total relative leaf nitrogen index.

### 3.6 Root length

The root length after 14 DAT at harvesting, in *M. piperita* in Group I, Group II, and Group III, showed an average (± SD) of 11.3 ± 0.6 cm, 10.3 ± 0.6 cm, and 11.0 ± 1.7 cm. Similarly in *C. annuum* in Group I, Group II, and Group III, the root length showed an average (± SD) of 13.3 ± 0.6 cm, 12.3 ± 1.2 cm, and 12.0 ± 1.0 cm. *G. globosa* in Group I, Group II, and Group III showed an average (± SD) of 11.0 ± 1.0 cm, 12.7 ± 0.6 cm, and 11.0 ± 1.0 cm.

### 3.7 Shoot fresh weight

**Figure 6:**
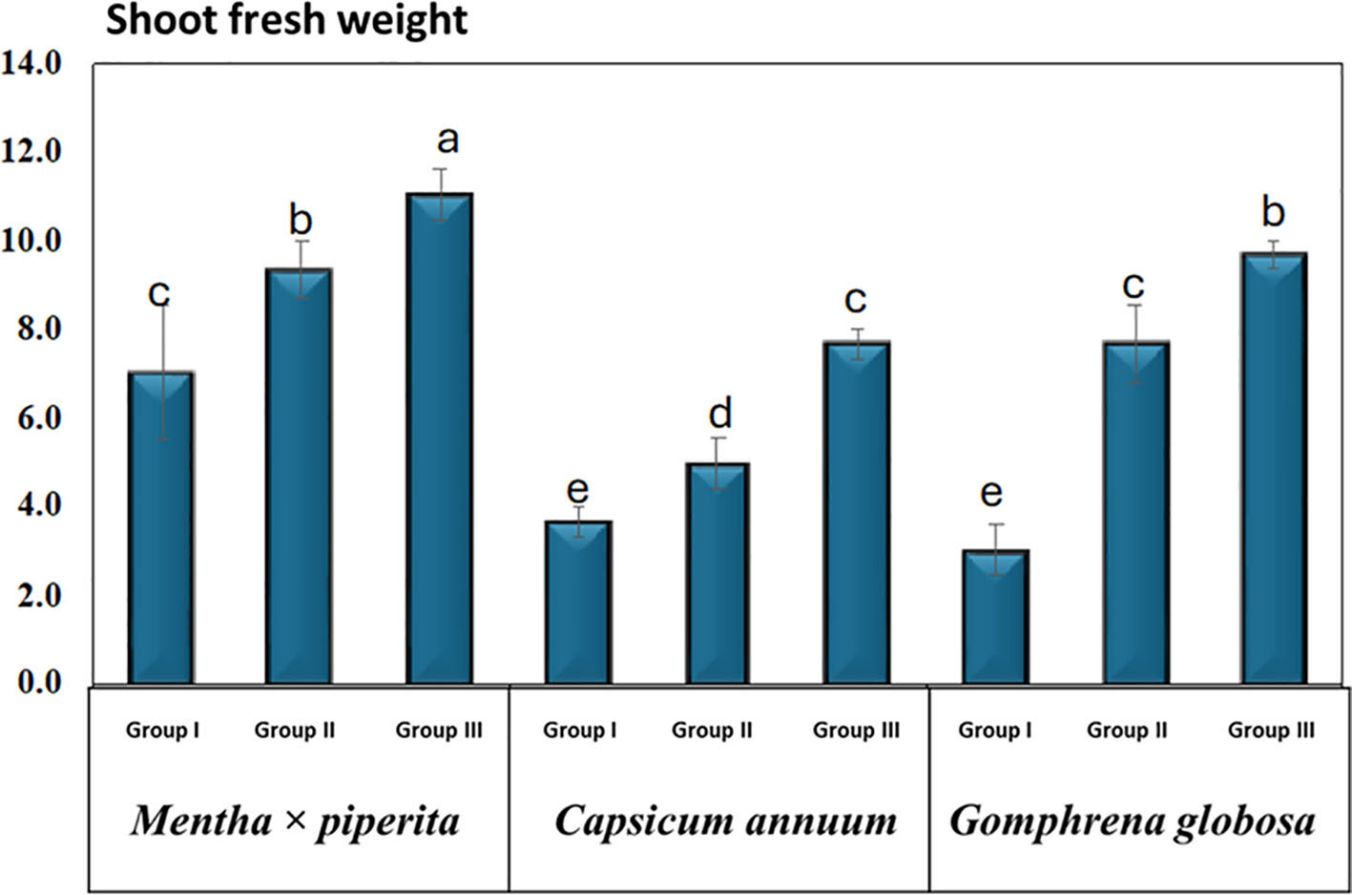
Shoot fresh weight under varying CO_2_ concentrations. Different letters indicate significant differences based on Tukey’s HSD test at *p* ≤ 0.05.

The shoot FW of the studied plant species was recorded at 14 DAT, corresponding to the harvesting stage, and is presented as mean ± SD (**Table 3**, **Figure 6**).

**Table 3:** The number of buds under varying CO_2_ concentrations presented as mean and standard deviation.

Treatment group	Shoot fresh weight		
	*Mentha × piperita*	*Capsicum annuum*	*Gomphrena globosa*
Group I	7.0 ± 2.6 c	3.7 ± 0.6 b	3.0 ± 1.0 c
Group II	9.3 ± 1.2 b	5.0 ± 1.0 b	7.7 ± 1.5 b
Group III	11.0 ± 1.0 a	7.7 ± 0.6 a	9.7 ± 0.6 a
Two-way repeated-measures ANOVA			
Plant species	<0.001***		
Treatment	<0.001***		
Plant × Treatment × Time	<0.001***		

Significance levels: *p* < 0.05 (*), *p* < 0.01 (**), *p* < 0.001 (***); “ns” indicates non-significant differences (*p* > 0.05). Different letters (a, b) denote significant differences according to Tukey’s HSD test at *p* ≤ 0.05. Abbreviations: ANOVA, analysis of variance; DAT, days after treatment. CO_2_ treatments: Group I (Control, ~400 ppm CO_2_), Group II (Elevated CO_2_, ~2,900 ppm CO_2_), and Group III (Highly Elevated CO_2_, ~5,400 ppm CO_2_).

For *M. piperita*, shoot FW in Group I was 7.0 ± 2.6 g, while in Group II and Group III treatment groups, the values increased to 9.3 ± 1.2 g and 11.0 ± 1.0 g, respectively. In *C. annuum*, shoot FW in Group I was measured at 3.7 ± 0.6 g, 5.0 ± 1.0 g in Group II, and 7.7 ± 0.6 g in Group III. Similarly, in *G. globosa*, shoot FW was 3.0 ± 1.0 g in Group I, while the treatment in Group II and Group III showed higher values of 7.7 ± 1.5 g and 9.7 ± 0.6 g, respectively.

### 3.8 Root fresh weight

**Figure 7:**
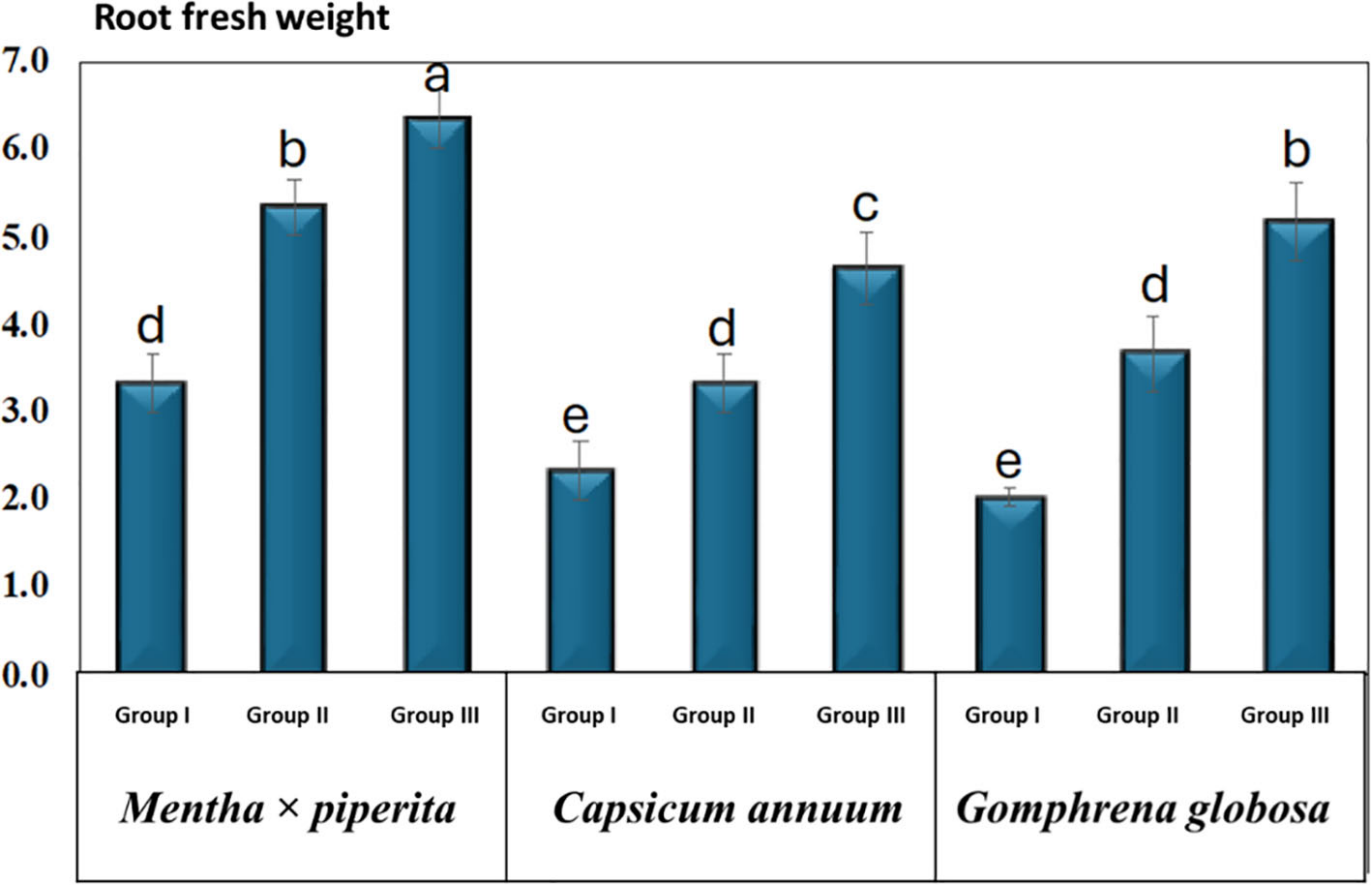
Root fresh weight under varying CO_2_ concentrations. Different letters indicate significant differences based on Tukey’s HSD test at *p* ≤ 0.05. Treatments included Group I (Control, ~400 ppm CO_2_), Group II (Elevated CO_2_, ~2,900 ppm), and Group III (Highly Elevated CO_2_, ~5,400 ppm). DAT, Days after treatment.

The root FW of the studied plant species was measured at 14 DAT, corresponding to the harvesting stage, and is presented as mean ± SD (**Table 4**, **Figure 7**). For *M. piperita*, the root FW in Group I was 3.3 ± 0.6 g, while the treatment groups in Group II and Group III exhibited increased values of 5.3 ± 0.6 g and 6.3 ± 0.6 g, respectively. In *C. annuum*, root FW was recorded as 2.3 ± 0.6 g in Group I, whereas Group II and Group III treatments resulted in higher values of 3.3 ± 0.6 g and 4.6 ± 0.7 g, respectively. Similarly, for *G. globosa*, root FW in Group I was 2.0 ± 0.2 g, with significant increases observed in Group II (3.7 ± 0.8 g) and Group III (5.2 ± 0.8 g) treatment groups.

**Table 4:** The plant’s root FW (g) under varying CO_2_ concentrations.

Treatment group	Root fresh weight		
	*Mentha × piperita*	*Capsicum annuum*	*Gomphrena globosa*
Group I	3.3 ± 0.6 a	2.3 ± 0.6 a	2.0 ± 0.2 a
Group II	5.3 ± 0.6 a	3.3 ± 0.6 a	3.7 ± 0.8 a
Group III	6.3 ± 0.6 a	4.6 ± 0.7 a	5.2 ± 0.8 a
Two-way repeated-measures ANOVA			
Plant species	<0.001***		
Treatment	<0.001***		
Plant × Treatment × Time	<0.001***		

Significance levels: *p* < 0.05 (*), *p* < 0.01 (**), *p* < 0.001 (***); “ns” indicates non-significant differences (*p* > 0.05). Different letters (a, b) denote significant differences according to Tukey’s HSD test at *p* ≤ 0.05. Abbreviation: ANOVA, analysis of variance. CO_2_ treatments: Group I (Control, ~400 ppm CO_2_), Group II (Elevated CO_2_, ~2,900 ppm CO_2_), and Group III (Highly Elevated CO_2_, ~5,400 ppm CO_2_).

### 3.9 Plant fresh weight

**Figure 8:**
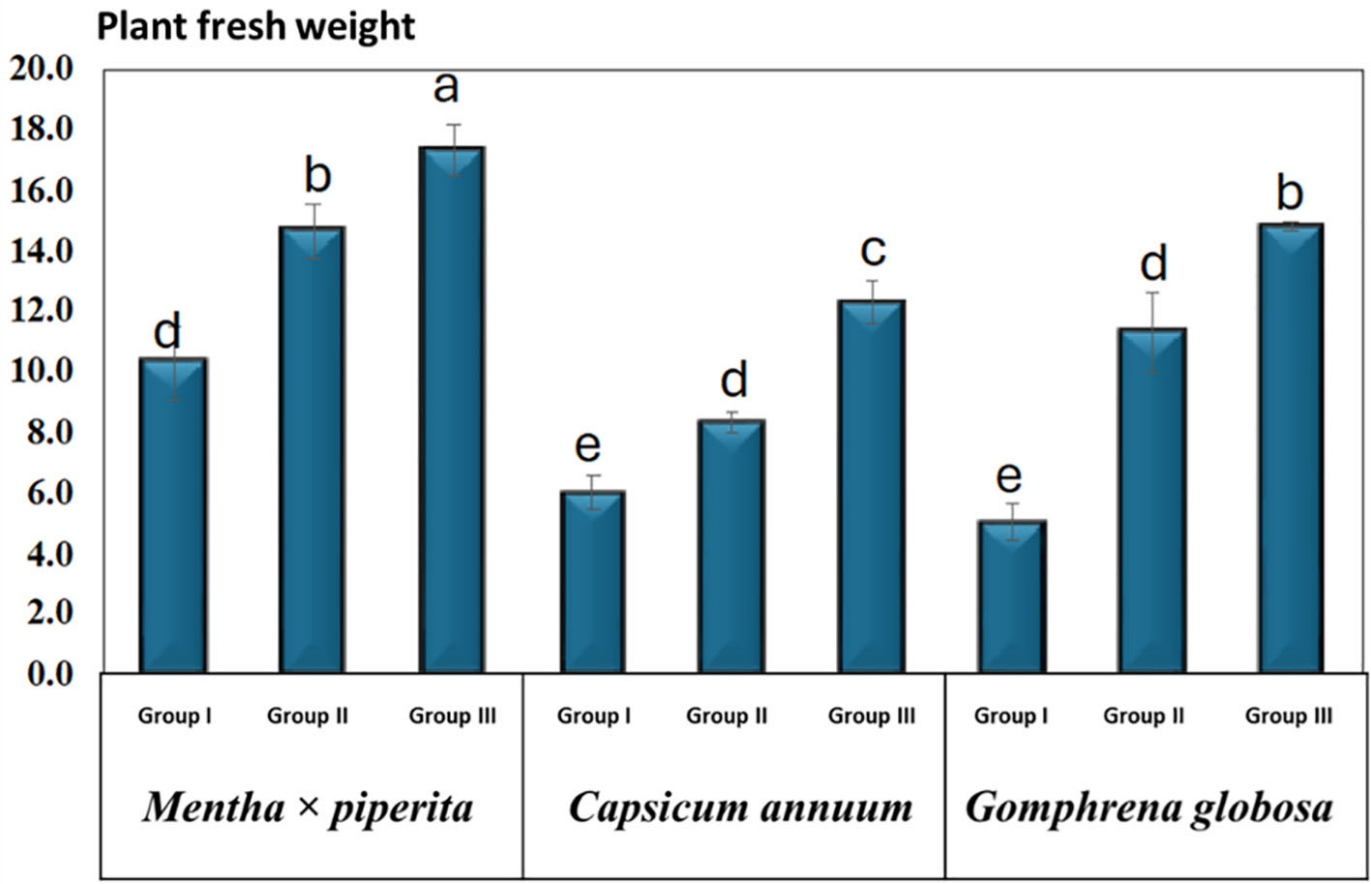
Plant fresh weight under varying CO_2_ concentrations. Different letters indicate significant differences based on Tukey’s HSD test at *p* ≤ 0.05. Treatments included Group I (Control, ~400 ppm CO_2_), Group II (Elevated CO_2_, ~2,900 ppm), and Group III (Highly Elevated CO_2_, ~5,400 ppm). DAT, Days after treatment.

The plant FW of the studied species was measured at 14 DAT, corresponding to the harvesting stage and is presented as mean ± SD (**Table 5**, **Figure 8**). For *M. piperita*, plant FW in Group I was 10.3 ± 2.1 g, while Group II and Group III treatment groups exhibited higher values of 14.7 ± 1.5 g and 17.3 ± 1.5 g, respectively. In *C. annuum*, plant FW Group I was recorded as 6.0 ± 1.0 g, whereas Group II and Group III treatments resulted in increased values of 8.3 ± 0.6 g and 12.3 ± 1.2 g, respectively. Similarly, for *G. globosa*, plant FW in Group I was 5.0 ± 1.0 g, with notable increases observed in Group II (11.3 ± 2.3 g) and Group III (14.8 ± 0.3 g) treatment groups.

**Table 5:** Plant FW (g) in *Mentha* × *piperita*, *C. annuum*, and *G. globose*.

Treatment group	Plant fresh weight		
	*Mentha × piperita*	*Capsicum annuum*	*Gomphrena globosa*
Group I	10.3 ± 2.1 c	6.0 ± 1.0 c	5.0 ± 1.0 c
Group II	14.7 ± 1.5 b	8.3 ± 0.6 b	11.3 ± 2.3 b
Group III	17.3 ± 1.5 a	12.3 ± 1.2 a	14.8 ± 0.3 a
Two-way repeated-measures ANOVA			
Plant species	<0.001***		
Treatment	<0.001***		
Plant × Treatment × Time	<0.001***		

Significance levels: *p* < 0.05 (*), *p* < 0.01 (**), *p* < 0.001 (***); “ns” indicates non-significant differences (*p* > 0.05). Different letters (a, b) denote significant differences according to Tukey’s HSD test at *p* ≤ 0.05. Abbreviations: ANOVA, analysis of variance; Plant FW, plant fresh weight. CO_2_ treatments: Group I (Control, ~400 ppm CO_2_), Group II (Elevated CO_2_, ~2,900 ppm CO_2_), and Group III (Highly Elevated CO_2_, ~5,400 ppm CO_2_).

### 3.10 Shoot/root ratio

The shoot-to-root ratio (shoot/root ratio) was assessed at 14 DAT, corresponding to the harvesting stage, and is presented as mean ± SD (**Table 6**). For *M. piperita*, the shoot/root ratio in Group I was 2.2 ± 1.1, while Group II and Group III treatment groups exhibited lower values of 1.8 ± 0.2 and 1.7 ± 0.1, respectively. In *C. annuum*, the shoot/root ratio in Group I was recorded as 1.6 ± 0.3, with similar values observed in Group II (1.6 ± 0.5) and Group III (1.7 ± 0.2) treatment groups. For *G. globosa*, the shoot/root ratio in Group I was 1.5 ± 0.5, whereas in Group II, it resulted in an increased ratio of 2.1 ± 0.2, followed by a slight decrease in Group III (1.9 ± 0.4).

**Table 6:** The shoot/root ratio (g.g^−1^) in plants.

Treatment group	Shoot/root ratio		
	*Mentha × piperita*	*Capsicum annuum*	*Gomphrena globosa*
Group I	2.2 ± 1.1 a	1.6 ± 0.3 a	1.5 ± 0.5 a
Group II	1.8 ± 0.2 a	1.6 ± 0.5 a	2.1 ± 0.2 a
Group III	1.7 ± 0.1 a	1.7 ± 0.2 a	1.9 ± 0.4 a
Two-way repeated-measures ANOVA			
Plant species	>0.05 ns		
Treatment	>0.05 ns		
Plant × Treatment × Time	>0.05 ns		

Statistical analysis of plant growth parameters under different CO_2_ treatments. “ns” denotes non-significant differences (*p* > 0.05). Different letters (a, b) indicate statistically significant differences according to Tukey’s HSD test at *p* ≤ 0.05. Abbreviation: ANOVA, analysis of variance. CO_2_ treatments: Group I (Control, ~400 ppm CO_2_), Group II (Elevated CO_2_, ~2,900 ppm CO_2_), and Group III (Highly Elevated CO_2_, ~5,400 ppm CO_2_).

### 3.11 Correlation analysis of CO_2_ levels and plant physiological responses

**Figure 9:**
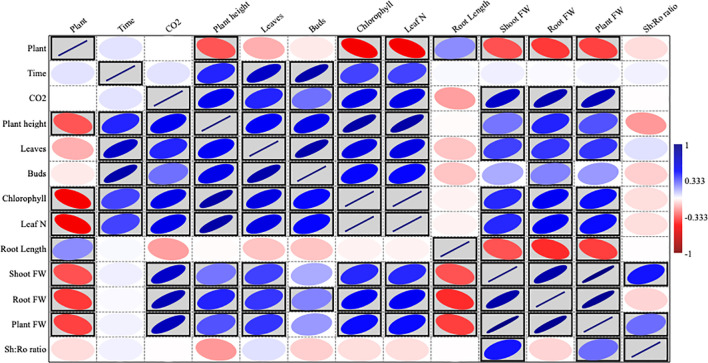
Blue–red heatmap illustrating the interrelationships between study variables. Blue indicates a positive correlation, red represents a negative (inverse) correlation, and white denotes no correlation. Gray boxes highlight statistically significant correlations.

The blue–red heatmap presented in **Figure 9** illustrates the correlation patterns among various study variables. In this heatmap, blue signifies a positive correlation, red represents a negative correlation, and white indicates no correlation. Statistically significant correlations are highlighted using gray boxes. The analysis reveals a strong inverse relationship between plant presence and CO_2_ levels, suggesting that the presence of plants significantly reduces CO_2_ concentration. Furthermore, indoor environments exhibited higher environmental factors (EFs) and noise levels compared to outdoor conditions. Additionally, a notable positive correlation was observed between CO_2_ levels and chlorophyll content, indicating that increased CO_2_ concentrations may influence photosynthetic pigment accumulation.

### 3.12 Canonical correspondence analysis of CO_2_ effects on plant growth

**Figure 10:**
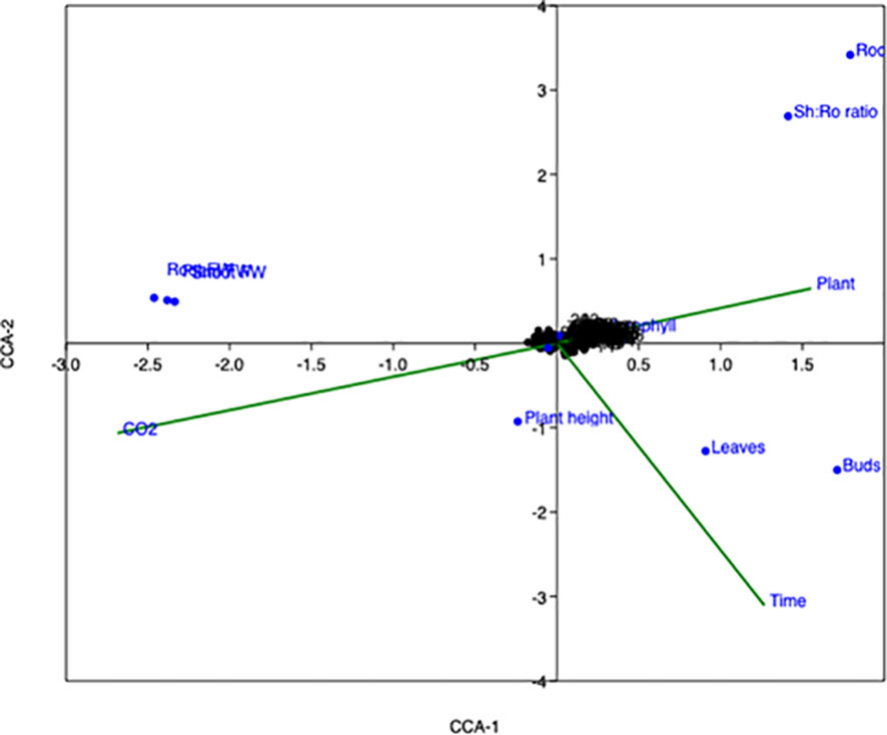
Canonical correspondence analysis (CCA) illustrating the interactions among study variables. Arrows represent the relationships between dependent and independent variables, with direction and length indicating the strength and significance of the associations.

The canonical correspondence analysis (CCA) presented in **Figure 10** illustrates the relationships between dependent and independent variables. In this multivariate analysis, arrows represent both the strength and direction of correlations, providing insights into how different variables respond to changes in CO_2_ levels and observation time. Independent variables, such as CO_2_ concentration and time of observation, are depicted by green arrows, with their length indicating the strength of their influence. The analysis demonstrates that CO_2_ concentration and observation time significantly impact chlorophyll content, total nitrogen, and plant growth parameters, highlighting the role of elevated CO_2_ in modulating physiological and biochemical processes in plants.

All measurements were conducted under standardized greenhouse conditions to ensure experimental reproducibility.

## 4 Discussion

The results of this study indicate that elevated CO_2_ levels significantly enhanced multiple physiological and biochemical traits, including plant height, leaf number, bud formation, biomass accumulation, chlorophyll content, and relative leaf nitrogen index in *C. annuum*, *G. globosa*, and *M. piperita*. At the highest CO_2_ concentration (5,400 ppm, Group III), *C. annuum* exhibited the most substantial growth response, displaying the most significant increase in plant height, number of buds, leaf chlorophyll content, and leaf nitrogen content, while also experiencing the most pronounced reduction in root length. It ranked second in leaf number and root weight increase but showed the smallest growth in shoot weight.

In contrast, *G. globosa* demonstrated the highest increase in leaf number, shoot weight, and root weight, with root length remaining unchanged. However, it exhibited the lowest response in all other measured parameters. *M. piperita* displayed a moderate response across most parameters under 5,400 ppm CO_2_, except for root weight, which showed the least increase among the three species. Overall, all three species experienced enhanced growth compared to Group I (ambient CO_2_ at 400 ppm), confirming that elevated CO_2_ positively affects plant growth parameters.

Elevated CO_2_ consistently enhanced growth-related parameters across the studied species, including plant height, leaf number, bud formation, shoot and root fresh weight, and total biomass. These findings are consistent with previous reports demonstrating that CO_2_ enrichment stimulates carbon assimilation and biomass accumulation in diverse crop species (Idso et al., 1987; Thompson et al., 2017; Yadav et al., 2019; Lamichaney et al., 2021; Mndela et al., 2022; Kaur et al., 2023). However, the magnitude of growth stimulation differed among species, with *C. annuum* exhibiting the strongest overall response at 5,400 ppm. Such variation likely reflects differences in photosynthetic pathway characteristics, resource allocation strategies, and physiological plasticity under high CO_2_ conditions.

Nutrient availability may further modulate plant responses to elevated CO_2_. Previous studies have shown that phosphorus limitation can reduce the stimulatory effects of CO_2_ enrichment on biomass accumulation (Ellsworth et al., 2017; Jiang et al., 2020; Li et al., 2023). Although nutrient levels were controlled in the present study, interactions between CO_2_ enrichment and nutrient dynamics remain important factors influencing long-term plant performance.

Regarding chlorophyll content, CO_2_ enrichment is crucial in modulating photosynthetic pigments. For instance, a study on wheat cultivars under drought stress demonstrated that elevated CO_2_ influences leaf photosynthesis and chlorophyll fluorescence parameters. These findings suggest that CO_2_ enrichment can alter chlorophyll content and photosynthetic efficiency, although the extent of these effects varies depending on plant species and environmental conditions (Yang et al., 2023). Although photosynthetic rate, stomatal conductance, and transpiration were not directly quantified in the present study, the observed increases in chlorophyll content and biomass under elevated CO_2_ are consistent with enhanced carbon assimilation. Elevated CO_2_ typically increases photosynthetic rate in C3 species by reducing photorespiration, while simultaneously decreasing stomatal conductance and transpiration, thereby improving water-use efficiency (Bravdo and Canvin, 1979; Engineer et al., 2016; Xu et al., 2016; Hatfield and Dold, 2019; Cao et al., 2022; Wei et al., 2022; Haghpanah et al., 2024). In C4 species, which already possess a CO_2_-concentrating mechanism, the stimulation of photosynthesis is generally less pronounced. Therefore, the growth enhancement observed in this study likely reflects improved carbon fixation efficiency and altered stomatal regulation under high CO_2_ conditions.

Our study demonstrated that elevated CO_2_ significantly enhanced chlorophyll and leaf nitrogen content, suggesting improved photosynthetic capacity. These findings are consistent with previous studies that reported increased photosynthetic rates and higher chlorophyll content under CO_2_ enrichment (Cai et al., 2021; Zhang et al., 2022). However, other research has documented decreased relative leaf nitrogen index under similar conditions (Laza et al., 2021). Additionally, Wang et al. (2023) found that elevated CO_2_ increases nitrogen concentrations in shoots while simultaneously reducing nitrogen levels in roots (Wang et al., 2023). Similarly, our study showed an overall increase in total relative leaf nitrogen index under elevated CO_2_ conditions (Wang et al., 2023).

The observed differences in relative leaf nitrogen index between C3 (*C. annuum* and *M. piperita*) and C4 (*G. globosa*) species may be partially explained by their contrasting photorespiratory pathways. In C3 plants, elevated CO_2_ reduces photorespiration, thereby improving nitrogen-use efficiency and potentially enhancing nitrogen accumulation in leaves. In contrast, C4 plants possess a CO_2_-concentrating mechanism that minimizes photorespiration even under ambient conditions, resulting in relatively smaller changes in nitrogen assimilation under elevated CO_2_. This physiological distinction may explain the comparatively moderate nitrogen response observed in *G. globosa.* The contrasting responses observed between the C3 and C4 species under 2,900 and 5,400 ppm further support the concept that C3 plants benefit more strongly from elevated CO_2_ due to reduced photorespiration, whereas C4 plants exhibit comparatively moderate stimulation because of their inherent CO_2_-concentrating mechanism. From an agricultural perspective, these findings suggest that C3 crops may exhibit stronger nitrogen-related growth stimulation under elevated CO_2_, whereas C4 crops may maintain more stable nitrogen metabolism due to their inherently efficient carbon fixation pathway.

The positive effects of CO_2_ enrichment on biomass accumulation have been widely documented. For instance, previous studies reported that elevated CO_2_ significantly increased tiller number, root dry weight, shoot dry weight, and total plant dry weight, even in the presence of cadmium stress (Jia et al., 2011). These findings align with our results, where elevated CO_2_ increased root and shoot FW across the tested species.

CO_2_ enrichment has also been shown to enhance plant height, with studies reporting increases of 4%–8% and 8%–29% during the flowering and podding stages, respectively (Lamichaney et al., 2021). Our study agreed with these findings and found that elevated CO_2_ promoted plant height. However, in contrast to previous research, we observed a reduction in root length under CO_2_ enrichment across the tested species, except for *G. globosa*, where root length remained unchanged. Additionally, our findings suggest that *G. globosa* exhibited enhanced growth under elevated CO_2_ conditions, aligning with the results reported by Silva et al. (2024). Previous research has shown that elevated atmospheric CO_2_ enhances photosynthesis and growth in amaranth. However, grain yield remains unaffected, protein content declines, and there is an increase in secondary metabolites and stress tolerance (Silva et al., 2024). These findings reinforce that CO_2_ enrichment can stimulate plant growth, but the overall physiological and metabolic responses depend on species-specific traits. Both our study and that of Pereyda-González et al. (2022) indicate that CO_2_ enrichment can enhance plant growth, although its effects are temperature-dependent (Pereyda-González et al., 2022). In our study, *C. annuum* exhibited the highest growth and chlorophyll content at 5,400 ppm CO_2_, suggesting a strong positive response to elevated CO_2_ levels. Similarly, previous research has demonstrated that *C. annuum* experiences improved seedling growth at moderate temperatures (30 °C). However, at higher temperatures (40 °C), CO_2_ enrichment fails to mitigate heat stress, impairing fruiting and development (Pereyda-González et al., 2022).

Our findings also align with previous research on *M. piperita*, where CO_2_ enrichment (620 ppm) increased biomass production by 48% and enhanced essential oil yield. Although our study tested higher CO_2_ concentrations (2,900 and 5,400 ppm), the observed growth stimulation supports the idea that *M. piperita* is exceptionally responsive to CO_2_ enrichment (Al Jaouni et al., 2018).

Similarly, a study on sweet pepper (*C. annuum*) found that short-term exposure to elevated CO_2_ (800 mmol mol^-1^) increased photosynthetic rates and biomass accumulation. However, long-term exposure led to acclimation, reducing photosynthetic efficiency after 135 days (Porras et al., 2017). Kumari et al. (2016) also reported that CO_2_ enrichment (550 ppm) combined with high temperatures improved yield attributes but reduced photosynthetic efficiency due to acclimation (Kumari et al., 2016). However, our study suggests that photosynthetic efficiency remains enhanced even at higher CO_2_ levels as indicated by increased chlorophyll content and plant biomass. This discrepancy may be attributed to differences in experimental conditions, such as controlled environments versus field studies (Karami et al., 2023). A notable trade-off observed in multiple studies is the dilution effect on nitrogen content (Dong et al., 2018; Yang et al., 2023).

While CO_2_ elevation enhances biomass accumulation, it can also lead to a decline in total leaf nitrogen content, a key factor influencing photosynthetic enzyme activity. This suggests that although elevated CO_2_ initially boosts growth, long-term plant responses may be constrained by nitrogen availability, potentially affecting overall nutritional quality (Andrews et al., 2019). This effect is particularly evident in crop species, where acclimation effects and eventual nitrogen limitations follow early increases in photosynthetic efficiency (Porras et al., 2017). Additionally, CO_2_ enrichment may reduce the concentration of essential nutrients, impacting the nutritional quality of crops. These findings underscore the importance of adopting a holistic approach when evaluating the impacts of elevated CO_2_ on agriculture and food security (Dong et al., 2018; Yang et al., 2023). This discussion highlights that while CO_2_ enrichment influences plant growth, its effects must be assessed within the broader context of environmental interactions, ensuring a comprehensive understanding of future agricultural challenges (Gonzalez-Meler et al., 2004; Jeong et al., 2018; Boretti and Florentine, 2019).

Future studies evaluating protein-to-carbohydrate ratios and carbon–nitrogen balance would provide deeper insight into metabolic adjustments under elevated CO_2_ conditions.

It should be noted that the present experiment evaluated short-term responses (14 DAT) under controlled conditions. Longer-term studies extending to full developmental stages and harvest would be necessary to determine the sustained effects of elevated CO_2_ on yield and reproductive performance. Future studies should include intermediate CO_2_ concentrations (e.g., 600–1,200 ppm) that better represent projected atmospheric scenarios, in order to assess gradual physiological and yield-related responses under more realistic climate conditions.

## 5 Conclusion

This study demonstrates that elevated CO_2_ levels significantly enhance plant growth and photosynthetic efficiency in *C. annuum*, *G. globosa*, and *M. piperita*. The observed growth stimulation can be attributed to improved water-use efficiency, enhanced RuBisCO activity, and increased photosynthetic capacity. However, the magnitude of these effects varied among species, highlighting the species-specific nature of plant responses to CO_2_ enrichment.

Despite the overall positive impact of elevated CO_2_, variations in photosynthetic efficiency, biomass allocation, and nutrient assimilation underscore the need for further investigations. Future research should focus on understanding the long-term physiological and biochemical adaptations of plants under CO_2_ enrichment, particularly in the context of climate change and global food security. Additionally, exploring the interactions between elevated CO_2_, nutrient availability, and abiotic stress factors will be crucial for developing sustainable agricultural strategies to mitigate potential challenges associated with rising atmospheric CO_2_ concentrations.

## Data Availability

The original contributions presented in the study are included in the article/**Supplementary Material**. Further inquiries can be directed to the corresponding author.
