# Supplementary material for: Comparative study on the effect of elevated levels of carbon dioxide on growth and photosynthesis of three selected plant species
Source: Front Plant Sci. 2026 May 15;17:1772837. doi: 10.3389/fpls.2026.1772837 (PMC13220251; doi:10.3389/fpls.2026.1772837)
Supplement: Supplementary file 1 [file DataSheet1.pdf]

Supplementary figures

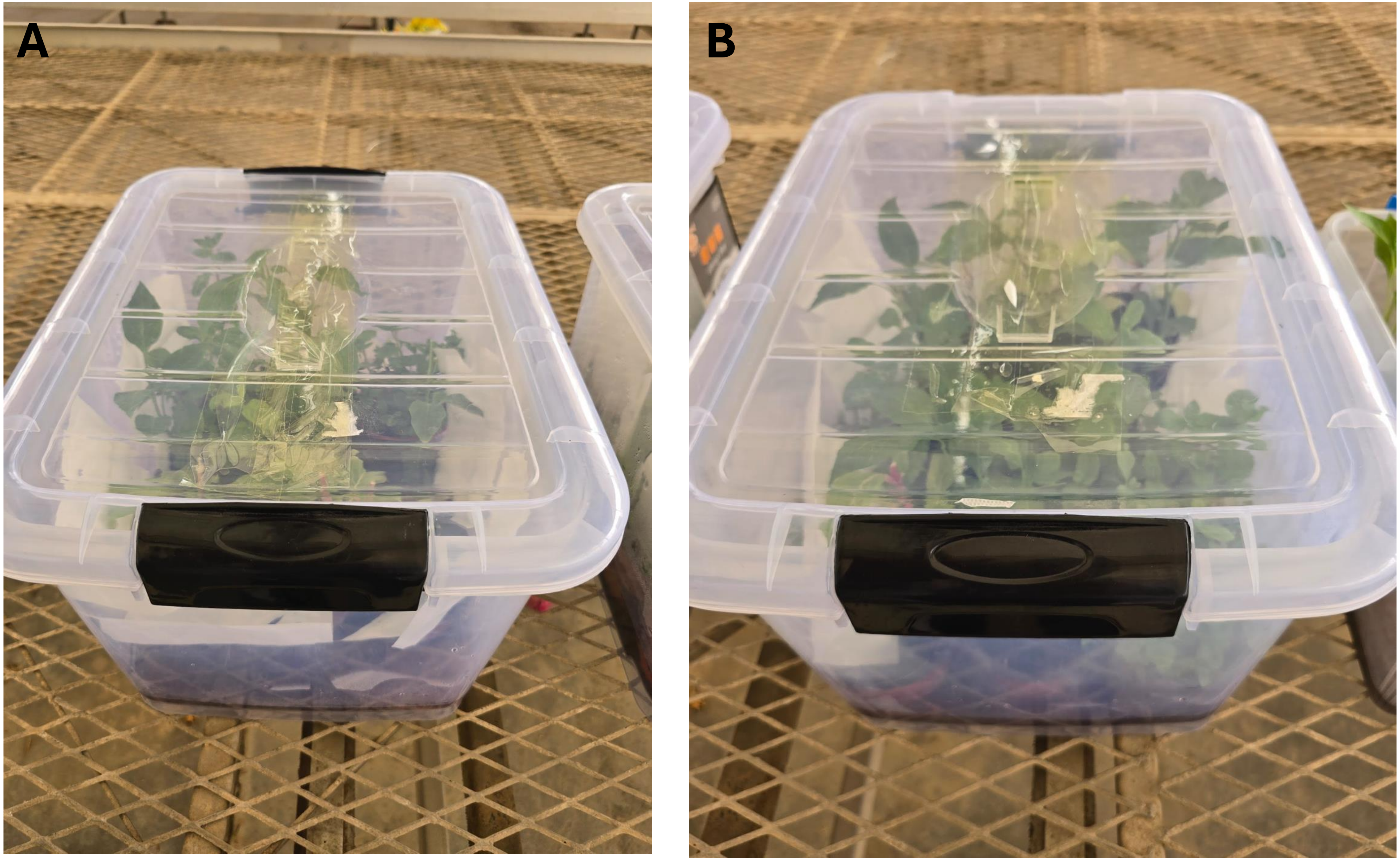

S-Figure 1. (A-C) Some selected experimental photos.

CO_2 Experimental Harvesting

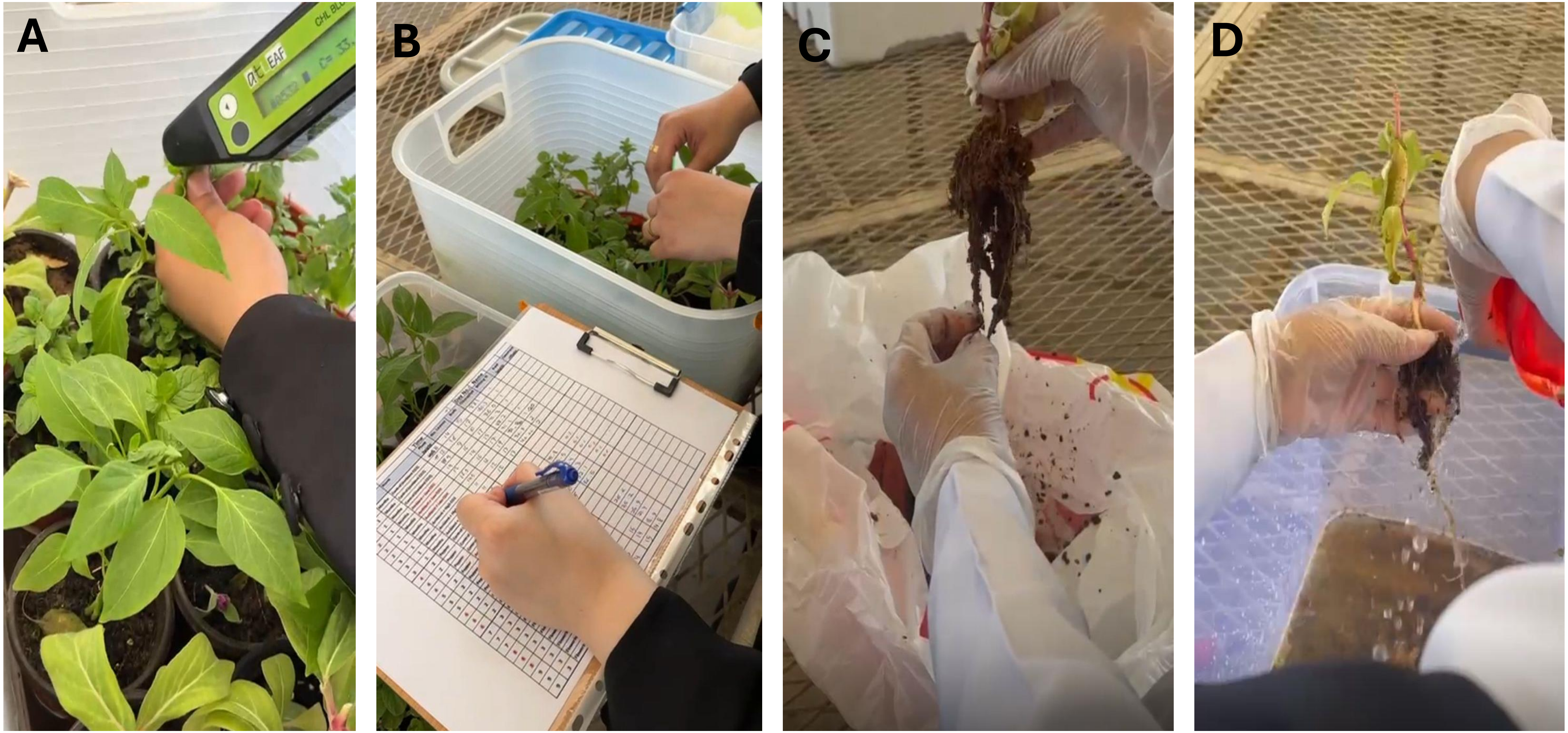

S-Figure 2. (A) Measuring chlorophyll content using Portable chlorophyll meter Atleaf. (B) recording various growth parameters, (C) Harvesting of experimental plants, washing root system

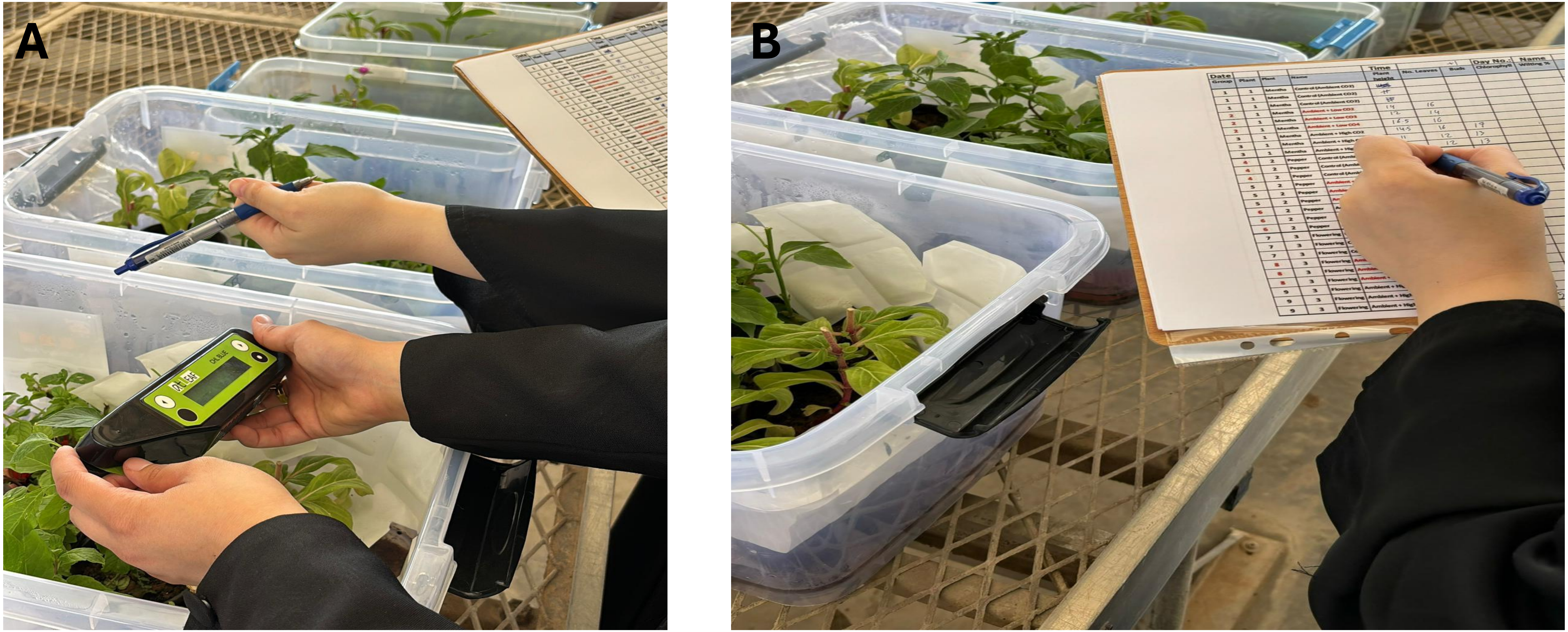

S-Figure 3. (A) Measuring chlorophyll content using Portable chlorophyll meter Atleaf. (B) recording various growth parameters.

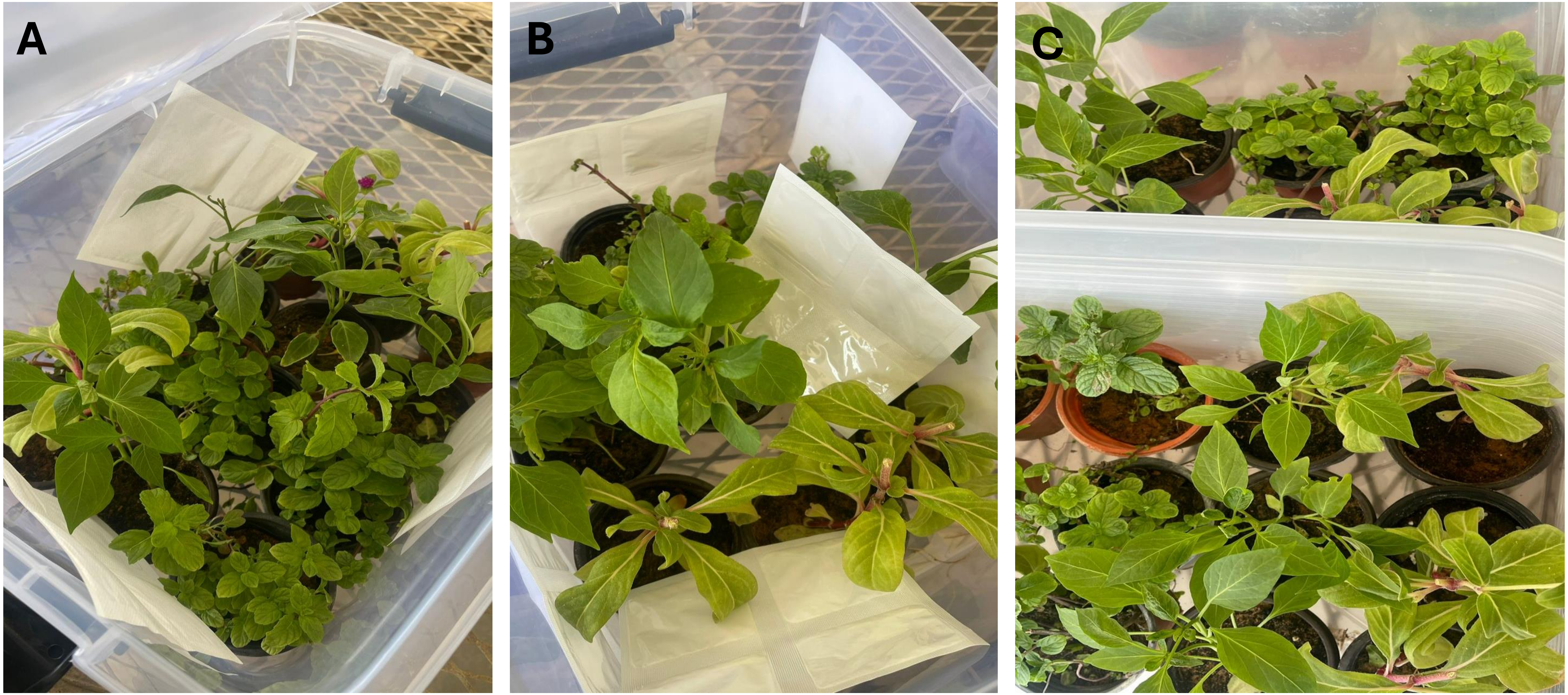

S-Figure 4. (A-C) Some selected experimental photos.
